# Clinically Meaningful Improvements in Long COVID Symptoms Following Ketogenic Metabolic Therapy Combined with Lifestyle Interventions—A Clinical Case Report and Review of the Literature

**DOI:** 10.4236/crcm.2025.148052

**Published:** 2025-08-06

**Authors:** Dana Dharmakaya Colgan, Diane D. Stadler, Aluko A. Hope, Heather Zwickey, Todd E. Davenport, Thomas Weimbs

**Affiliations:** 1 Helfgott Research Institute, National University of Natural Medicine, Portland, OR, USA; 2 Department of Neurology and Long Covid Clinic Program, Oregon Health & Science University, Portland, OR, USA; 3 School of Medicine, Oregon Health & Science University, Portland, OR, USA; 4 Division of Pulmonary, Allergy and Critical Care Medicine, School of Medicine, Oregon Health & Science University, Portland, OR, USA; 5 Department of Physical Therapy, School of Health Sciences, University of the Pacific, Stockton, CA, USA; 6 Department of Molecular, Cellular, and Developmental Biology, University of California Santa Barbara, Santa Barbara, CA, USA; 7 Santa Barbara Nutrients, Inc, Santa Barbara, CA, USA

**Keywords:** Long COVID, Infection-Associated Chronic Illness, Ketogenetic Metabolic Health, Myalgia Encephalomyelitis/Chronic Fatigue Syndrome, Dysautonomia

## Abstract

Approximately 400 million individuals globally are estimated to suffer from Long COVID, an infection-associated chronic condition that occurs after SARS-CoV-2 infection. Despite the high burden, there are no evidence-based or FDA-approved interventions to treat the condition. Given its complexity, a multicomponent approach grounded in a whole-person health model is likely required. This case report highlights clinically meaningful improvements in multiple Long COVID symptoms following a remotely-delivered, ketogenic metabolic therapy combined with group health coaching. Nutritional interventions were paired with exercises to stabilize circadian rhythms and introduce mindfulness-based practices. A review of the literature provides evidence in support of ketogenic metabolic therapy and lifestyle interventions as strategies to target proposed underlying mechanisms of Long COVID and foster stress resilience, thus reducing symptoms and improving quality of life. Findings support future research to optimize and evaluate multimodal nutritional and lifestyle interventions for Long COVID.

## 1. Introduction

Long COVID (LC) is a chronic condition that develops after a SARS-CoV-2 infection and persists for at least three months. It presents as a continuous, relapsing-remitting, or progressive disease that affects one or more organ systems [1]. Approximately 400 million individuals globally are estimated to suffer from LC, leading to an annual economic impact of $1 trillion [2]. Nearly 70% of LC patients develop dysautonomia, a term describing various conditions involving maladaptive autonomic nervous system (ANS) responses [3]. Dysfunction of the ANS leads to abnormal compensatory responses, gastrointestinal symptoms, extreme fatigue, labile blood pressure, orthostatic hypotension, and dysfunction in heart rate variability. Further, approximately 58% of patients with LC develop myalgia encephalomyelitis/chronic fatigue syndrome (ME/CFS) [4], a complex, multisystem neurological disease. ME/CFS includes the dysregulation of the nervous, immune, and endocrine systems with impaired cellular energy metabolism and is characterized by its clinical hallmark of post-exertional malaise (PEM). PEM presents as a constellation of debilitating fatigue, unrefreshing and disordered sleep, cognitive impairment, orthostatic intolerance, sensory sensitivity, amplified pain, recurrent headaches, and gastrointestinal disturbances in response to primarily physical and cognitive exertion [5] [6].

Despite the high burden of LC, no evidence-based or FDA-approved treatment exists [7]. A ketogenic diet is a high-fat, low-carbohydrate, and moderate-protein diet that has gained popularity in recent years in the context of neurological diseases [8]. This case report uses the 2012 CARE framework [9] to describe the experience of a patient with LC presenting with post-COVID dysautonomia and ME/CFS and the effects of a ketogenic metabolic therapy (KMT) and lifestyle intervention on multiple symptoms of LC. The patient provided consent for the development of this clinical case report and publication and the Oregon Health & Science University (OHSU) Institutional Review Board responded to a determination of research notice that a full IRB review was not necessary.

## 2. Case History/Examination

The patient was a 44-year-old, White, athletic, English-speaking female who completed post-graduate education. Medical history prior to 2020 was unremarkable. She presented at the OHSU Long COVID Clinic in December of 2023 with extreme fatigue, fatigue following cognitive and physical exertion, nausea, daily headaches, shortness of breath, chest pain, and tachycardia.

In February 2020, she developed a viral upper respiratory infection characterized by several days of a high fever (103 - 104°F), chills, cold sweats, fatigue, and upper respiration symptoms. In March of 2020, the patient presented to her primary care physician describing changes in cognitive function, specifically in domains of attention, vigilance, and executive functioning, along with intermittent phonetic and semantic paraphasia. She was referred to the department of psychiatry that ruled out depression. No COVID-19 testing was available, however, before her first COVID-19 vaccination in January of 2021, she tested positive for SARS-CoV-2 antibodies. Findings supported the suspicion that the February 2020 infection was COVID-19. Within eight hours of receiving her first dose of a Pfizer COVID-19 mRNA vaccine, she experienced bilateral swelling and excruciating neuropathic pain in hands and fingers, severe and diffuse joint pain, migratory rashes, blisters below her fingernail beds, hot and rough tongue, “COVID toes” that appeared bright red, swollen lymph nodes, nausea, chills, fatigue, muscles aches, headaches, brain fog, dizziness, tingling, and vertigo. In March 2021, she was diagnosed with a multisystem inflammatory syndrome by an infectious disease specialist. She subsequently tested positive for COVID-19 four more times (01/2022, 08/2022, 11/2022, and 08/2023), with acute symptoms including fevers, chills, cough, myalgia, brain fog, joint and nerve pain, and anosmia. Symptoms lasted 7 - 10 days. She was treated with a five-day course of Paxlovid (nirmatrelvir/ritonavir) after the third, fourth, and fifth infections.

Eight months after her fourth infection, she developed worsening post-exertional fatigue. She described episodes of severe fatigue where she could not move or open her eyes for 20 - 30 minutes, followed by several days of bed-bound exhaustion. Other symptoms included weight gain, nausea, daily headaches, chest pain, tachycardia, light and sound sensitivity, flushing, and chronic dry eyes and mouth. Flushing episodes lasted 15 - 40 minutes and occurred after eating, walking, or cognitive/social activity, often with palpitations and shortness of breath. She reported irregular uterine bleeding. The patient was regularly hypotensive, with systolic and diastolic blood pressures of approximately 80/50, with a resting heart rate of 119. Serum ferritin concentration was 13 ng/mL, with normal serum iron and hemoglobin values. By December 2023, she was placed on medical leave, during which time she was unable to bathe, cook, shop, or walk independently.

**Figure 1.**
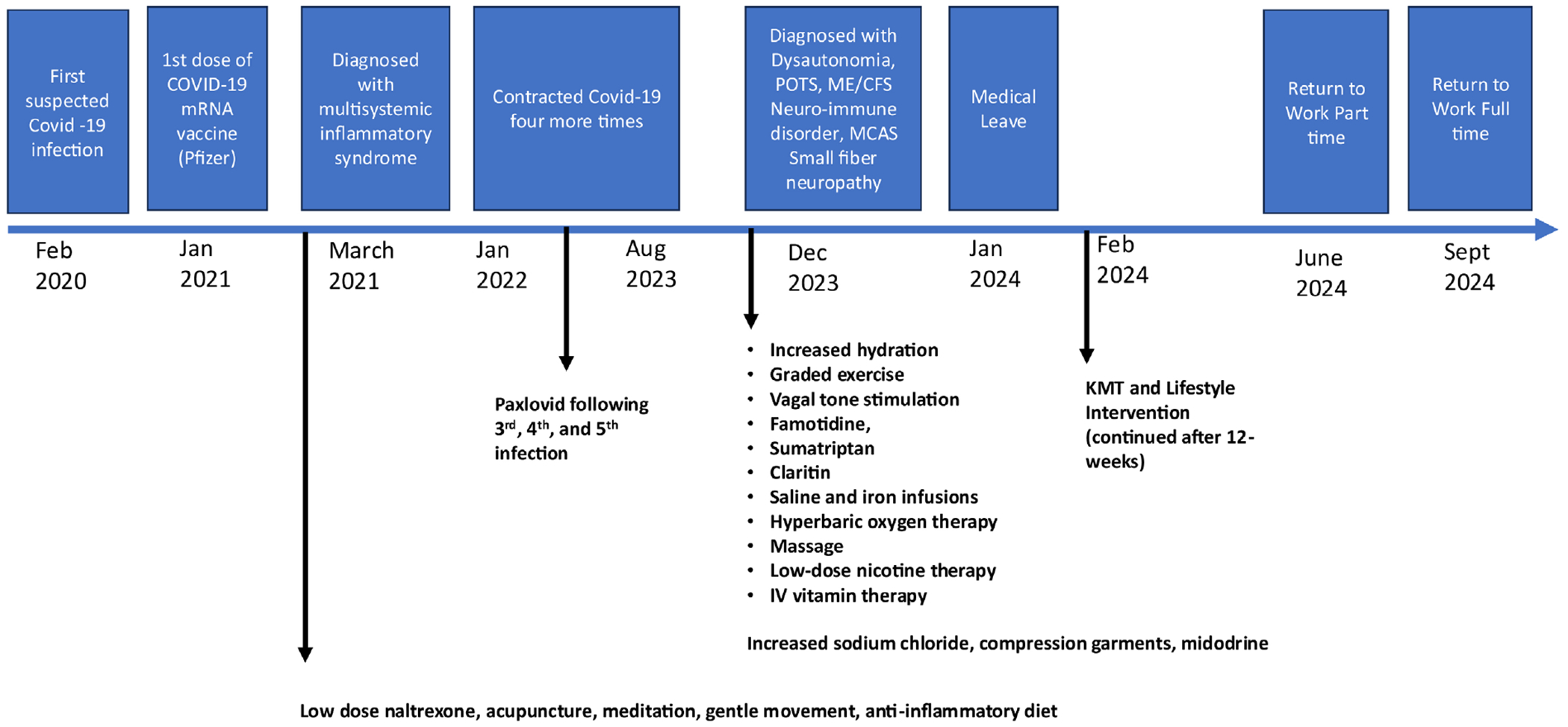
Timeline of corresponding treatments.

Starting in November 2023, she trialed multiple treatments. Treatment recommendations from the Long COVID clinic included, increased oral sodium chloride (3000 mg/daily), increased hydration (3 - 4 liters/daily), compression garments, and pacing. Treatment approaches recommended by functional medicine physicians included, low-dose nicotine therapy and IV vitamin therapy [*i.e.*, glutathione, vitamin C, Myer’s Cocktail (B-1, B-2, B3, B5, B6, B12, Vit C, Magnesium, trace elements)]. She also attempted vagal tone stimulation, acupuncture, meditation and mindful movement, massage, and followed an anti-inflammatory diet, which closely resembled a paleo diet (Wahls Paleo) [10]. Prescription medications included low dose naltrexone (4.5 mg/ daily) for immune regulation and pain, guanFACINE (1 mg/daily) for cognition and autonomic regulation, antihistamines (*i.e.*, famotidine-40 mg/daily and Claritin-20 mg/daily) to reduce flushing episodes, midodrine (2.5 mg 3 - 4 times a day) for vasoconstriction, and sumatriptan for migraines (2 - 4 times a week, or as needed). Iron infusions resolved iron deficiency defined by low ferritin concentration. Low dose naltrexone decreased neuropathic pain. Bi-weekly saline infusions reduced hypotension for 24 - 36 hours, but normotension was not maintained. Hyperbaric oxygen therapy, graded exercise, and massage was followed by severe PEM. Information from these episodes of care is organized in Figure 1.

## 3. Differential Diagnosis, Investigations, and Treatment

Diagnostic assessments are presented in Table 1. Patient-reported outcomes were measured using standardized tools and are shown in Table 2. The patient was diagnosed with post-viral dysautonomia, specifically Postural Orthostatic Tachycardia Syndrome (POTS), based on results from a tilt table test. POTS is a dysfunction of the autonomic nervous system that involves symptoms triggered or worsened by standing, such as dizziness, fainting, headaches, shortness of breath, and a heart rate increase of at least 30 beats per minute when moving from lying to standing. She was also diagnosed with ME/CFS with severe physical and cognitive fatigue and PEM and a neuro-immune disorder. Mast cell activation syndrome was a probable diagnosis, but unconfirmed. A skin biopsy revealed small fiber neuropathy, evidenced by abnormal nerve fiber density at distal sites. Objective investigations ruled out reactivation of the Epstein-Barr, herpes, and parvovirus viruses, systematic lupus, Sjogren’s syndrome, Graves’ Disease, celiac disease, and myasthenia gravis. The clinical progression included a documented vaccine injury, followed by multiple COVID-19 infections, and the eventual onset of clinical symptoms and diagnoses of LC and associated chronic illness, such as POTS and ME/CFS. While there is a clear need to distinguish between post-acute COVID-19 vaccination syndrome and LC, the lack of scientific and clinical data impeded the ability to discriminate between to the diagnoses [11] [12].

In February of 2024, the patient enrolled in a community-based 12-week educational program designed for dysautonomia, termed “*Enable Your Healing,*” that combined KMT with lifestyle components. The virtual, 12-week nutrition and educational program was conducted using weekly remote video conference calls. This program used a specialized form of the ketogenic diet comprised of low-carbohydrate, and moderate-protein intake to change the patient’s metabolic state from a carbohydrate-dependent glycolytic state to a fat-dependent ketogenic state. The lifestyle components of the program included daily activities to entrain circadian rhythm and mindfulness-based strategies to increase parasympathetic activation and foster psychological resilience. Medical nutritional therapists and health coaches provided the virtual intervention. Program details are displayed in Table 3.

## 4. Outcome and Follow-Up

Patient-reported outcomes were collected at the OHSU Long COVID and Physical Rehabilitation Clinics. Clinically meaningful change was assessed using the concept of minimal clinically important difference (MCID), which avoids overreliance on statistically significant results without clinical relevance. Clinical improvements following the intervention were identified in five of the seven patient-reported outcome measures across domains of dysautonomia, shortness of breath, migraine disability, fatigue, and physical function (Table 2) [13]-[20].

Within the first two weeks of the intervention, her chronic hypotension resolved, and she discontinued IV saline therapy. She reported a notable increase in energy that she reported coincided with the onset of ketosis. By the second month, sound and light sensitivity had resolved. Menorrhagia and irregular uterine bleeding ceased, and her menstrual cycle normalized to 25 - 29 days. Headaches reduced from 4 - 5 times per week to once or twice a month, typically linked to ovulation or menstruation. She no longer experienced extreme fatigue episodes and brain fog improved. Although she continued to experience PEM after moderate exertion, episodes of flushing were diminished and described as “less frequent and less severe.”

Over 12 weeks, she lost 15% of her body weight, reducing her BMI from 23 to 20 kg/m^2^. By the program’s end, she could independently bathe, cook, shop, and walk. She resumed cognitive work for 1 - 3 hours every other day. She continued the intervention after the program concluded. By month seven, she was working cognitively 5 - 6 hours per day and exercising 20 minutes daily on a recumbent bike without triggering PEM. That same month, she reintroduced fermented dairy and regained weight, stabilizing at a BMI of 21 kg/m.

Adherence to the KMT was assessed daily via blood ketone and glucose levels using a Mojo Keto device, (Keto-Mojo, 952 School Street 212, Napa, CA 94559). Fasting blood ketones ranged from .08 to 4.2 mmol/L. Fasting blood glucose levels ranged from 70 - 100 mg/dL. Macronutrients were recorded daily on the Carb Manager iPhone app. Average micronutrient intake was approximately 20 grams of carbohydrates, 60 grams of protein, and 150 plus grams of fat. Documentation of engagement in weekly lifestyle activities was recorded weekly. The patient attended 100% of nutrition conference calls, 100% of lifestyle calls, and 76% of mind-set calls. Laboratory results (*i.e.*, renal, hepatic, and electrolyte panels) were all within the normal reference range, supporting the safety of 2000 mg/day thiamine and 3000 mg magnesium in adults with dysautonomia.

## 5. Discussion

This clinical case report highlights potential benefits of combining ketogenic metabolic therapy with health coaching to reduce symptom burden and improve quality of life for individuals with LC. While the study design does not afford the examination of causal effects, findings are in stark contrast to results from a recent nested population-based case-control study which included 982 participants with Long COVID and 576 age- and sex-matched control subjects without Long COVID. Authors reported that within the second year of illness, the majority of working-age patients with Long COVID reported that symptoms remained essentially similar, non-specific, and dominated by fatigue, exercise intolerance and cognitive complaints [21]. Moreover, Davis *et al.* reported that only 27.3% of participants with current Long COVID syndrome symptoms had resumed their usual working hours, with 45.6% working reduced hours and 23.3% not working as a direct result of their symptoms [7]. KMT has shown promise in acute infections, including SARS-CoV-2, but these findings suggest that KMT in Long COVID and other IACIs may prove beneficial.

### Evidence for Ketogenic Metabolic Therapy

Several biological mechanisms of KMT may be relevant for LC prevention and treatment, including reduced circulating insulin and glucose and increased mitochondrial energy production [22]. There is a growing perspective that acute COVID-19 and LC are metabolic diseases. Studies have linked the degree of hyperglycemia to the severity of an acute SARS-CoV-2 infection, as high blood glucose concentrations promote viral replication, leading to cytokine production and T-cell dysfunction. A systematic review and meta-analysis, encompassing nine investigations with nearly 40 million participants, reported a significant correlation between COVID-19 infection and an increased risk of diabetes [23], and a prospective observational study found that 75% of patients with long COVID-19 developed diabetes within a year of acute infection with COVID-19 [24]. Moreover, Metformin use among COVID-19 patients is associated with a reduced risk of mortality and hospitalization [25], and when prescribed within a week of COVID-19 infection, metformin reduced long COVID incidence by about 41%, compared with placebo [26]. Exact mechanisms behind a higher risk of new-onset diabetes following COVID-19 infection are not yet clear, however, insulin resistance has been proposed as a central mechanism. Prolonged hyperglycemia, because of a virus-induced insulin resistance, may increase mitochondrial reactive oxygen species and reduce antioxidant defenses, leading to increased oxidative stress and a global proinflammatory state. Importantly, a large body of evidence has confirmed the effectiveness of the KMT in treating insulin resistance and diabetes by significantly reducing hyperglycemia and hyperinsulinemia [27]. Further, smaller, more recent studies, suggest that KMT could be used to blunt COVID-19 related hyperinflammation [28], may have a protective role against metabolic abnormalities after COVID-19 [29], and should be considered when treating patients with LC.

Another potential driver of chronic inflammation in LC is mitochondrial dysregulation and impaired redox signaling [30] [31]. Specifically, the SARS-CoV-2 virus RNA causes mitochondrial dysfunction, altering cellular energy production, which, in turn, increases oxidative stress, resulting in a loss of mitochondrial integrity and cell death [32] [33]. When viral proteins bind to mitochondrial complexes and disrupt mitochondrial function, they cause immune cells to overreact, contributing to chronic and systemic inflammation. This proinflammatory state, and the imbalanced immunoreaction, has been proposed to contribute to insufficient insulin synthesis and insulin resistance.

KMT may counteract mitochondrial dysfunction and inflammation by switching the primary mitochondrial fuel source from glucose to ketones [34]. During a prolonged restriction of carbohydrate intake, hepatically produced ketone bodies, β-hydroxybutyrate and acetoacetate, become the primary energy source for the mitochondria to produce adenosine triphosphate (ATP), the main energy source in the body. Ketone bodies bypass several key steps in glucose transport and glycolysis which are thought to be disrupted in acute COVID-19 and LC [35]. *β*-hydroxybutyrate then acts as both a highly efficient oxidative fuel and signaling metabolite to counteract the reduced ATP production and impaired brain energy metabolism by producing over 20% more ATP per molecule than glucose. KMT also decreases oxidative stress in the mitochondria [36] and has been shown in preliminary human studies to downregulate pro-inflammatory cytokines including IL-1*β*, IL-6, and TNF-alpha [37]. *β*-hydroxybutyrate has evidenced neuroprotective benefit to support brain health, inhibits the NLRP3 inflammasome, and demonstrates other potent anti-inflammatory effects [38] [39].

The patient reported that hypotension was one of the first symptoms to resolve and we can speculate that the KMT may have contributed. During ketosis, the body expels water and salt, creating a dehydrating effect. This often results in weight loss; however, it is possible, but not conclusive, that reduced blood volume may have also improved hypotension.

### Evidence for the Thiamine and Electrolyte Supplementation

Thiamine, also known as vitamin B1, is a precursor of the essential coenzyme thiamine pyrophosphate that is required for glucose metabolism [40]. Thiamine is also the rate limited cofactor for a set of mitochondrial enzymes that are central to oxidative metabolism and has essential roles in nerve myelination, neurotransmission, immune function, and sodium/potassium homeostasis. Moreover, thiamine availability determines whether and how much ATP is produced. Organ systems requiring the most ATP are particularly suspectable to variation in mitochondrial efficiencies (*i.e.*, brain, heart, muscle, gastrointestinal), where dysautonomia and ME/CFS present [41].

Thiamine deficiency has been linked with the risk and severity of acute COVID-19, shown to produce reversible damage to the mitochondria, and can potentially result in inadequate antibody responses; however, high doses of thiamine given to patients at the early stages of COVID-19 was shown to limit episodes of hypoxia and decrease hospitalization [42]. An in-vitro study found that high-dose thiamine lowers the cell’s pro-inflammatory response (T-helper cells, Th-17) believed to be associated with the COVID-19 cytokine storm and hyperinflammation [36]. Recently, results from a randomized control trial found that vitamin B1 administration can significantly reduce the duration of LC [43].

In addition to vitamin B1 supplementation, the patient also increased her daily intake of magnesium, potassium, and other electrolytes (Table 3). Individuals with dysautonomia are often chronically dehydrated due to the deregulation of vasopressin, leading to cellular dehydration [44]. Electrolytes are thought to promote cellular hydration. Further, magnesium plays an essential role in several enzymatic reactions and its deficiency has been associated with a chronic inflammation and/or oxidative stress [45]. Further, increased magnesium supplementation is thought to assist an individual transition into a ketogenic state, support lipid metabolism [46], and may also contribute to adaptive immunity [47]. Recommended doses of thiamine and electrolyte supplementation were higher than the recommended daily allowance (https://ods.od.nih.gov/HealthInformation/nutrientrecommendations.aspx).

### Adverse Reactions

The “keto flu” is a temporary collection of flu-like symptoms individuals might experience in the first week or two of starting KMT, as the host’s metabolic state shifts from a carbohydrate-dependent glycolytic to a fat-dependent ketogenic state. Other possible adverse reactions to KMT include constipation, insomnia, dehydration, low bone density (osteopenia), and kidney stones. Possible adverse reactions to the large dosing of B1 include nausea, stomach ache, kidney stones, constipation, excessive thirst, and urination. The patient did not report experiencing these possible adverse reactions to KMT or vitamin supplementation.

### Evidence for Lifestyle Interventions as an Adjunct Therapy

Patients with LC have a high prevalence of sleep disturbance. A review and meta-analysis involving 14,000 LC patients found that the overall pooled prevalence of sleep disturbance was 46% (95% CI: 38 - 54%). Subgroup analyses revealed pooled prevalence of poor sleep quality was 56% (95% CI: 47 - 65%) and insomnia was 38% (95% CI: 28 - 48%) [48]. Underlying drivers of sleep disturbance in LC are likely multifactorial and mechanisms have yet to be clearly identified; however, it is well-established that sleep disruption is associated with increased activity of the sympathetic nervous system and hypothalamic–pituitary–adrenal axis, metabolic alterations, and proinflammatory responses, all implicated in LC [49]. Further, recent studies suggest that immune and inflammatory responses following a viral infection could contribute to disruptions in the circadian rhythm [50] [51], the master biological clock based in the brain’s hypothalamus that regulates sleep-wake cycles. The primary function of the circadian rhythm is to coordinate biological processes so they occur at the correct time to maximize homeostasis. Circadian disruptions desynchronizes mechanisms that coordinate physiological activity across organ systems, impairing metabolism and energy homeostasis, immune function, resulting in fatigue, depression, autonomic instability, cold intolerance, orthostatic hypotension, cognitive disturbance, symptoms associated with ME/CFS, POTS and LC [52]-[60]. The evidence linking circadian rhythms to ME/CFS, POTS, and LC remains inconclusive; however, it is clear that sleep is an essential target for LC treatment, and there is growing consensus that entraining circadian rhythms may be beneficial.

Lifestyle modifications to entrain circadian rhythms have been shown to be effective in improving sleep. A meta-analysis with 40 studies examined the effects of light interventions on sleep quality within sleep disorders and neuropsychiatric populations. Results indicated improvements in sleep continuity, self-reported sleep disturbance, and increased total sleep time [61]. Exposure to bright morning light stabilizes circadian timing, and may be beneficial in managing glucose metabolic disorders [62], and reducing depressive symptoms, fatigue, and pain in populations with fibromyalgia [63] and cancer [64].

Patients with LC also report high rates of stress, depression, and anxiety [65], [66] driven by various biologic and psychosocial factors including, but not limited to, neuroinflammation [67], stress of a chronic illness, experiences of stigmatization and feeling dismissed by medical providers, living with uncertainty and fear, inability to access effective medical treatment, and loss of previous physical, social, economic functioning [68]. Chronic stress, often defined as the response to emotional pressure suffered for a prolonged period of time in which individuals perceive that they have no control, can influence the pathogenesis of physical disease by causing negative affective states (e.g., feelings of anxiety and depression). Negative affective states in turn exert direct effects on biological processes or behavioral patterns that influence disease risk [69].

Meditation and mindfulness practices focus on training attention and non-reactive awareness to improve self-regulation and foster mental and physical well-being. Systematic reviews and meta-analyses have documented a consistent effect of mindfulness-based interventions (MBIs) on improving patient-reported outcomes, including symptoms of stress, anxiety, depression, pain, and quality of life [70]-[72], as well as reducing physiological markers of stress [73]. In populations with pre-COVID ME/CFS, MBIs have been shown to significantly reduce fatigue severity, pain, depression, and anxiety, improve quality of life and immune function, down-regulate sympathetic autonomic functioning to promote a more restful resting state, and have shown anti-inflammatory properties, when compared to wait-list or support group controls [74]. However, small sample sizes and heterogeneous diagnostic criteria have limited our understanding of the impact on MBIs in this population. MBIs also show evidence to improve quality of sleep. A systematic review and meta-analysis that included 18 trials with 1654 participants with disturbed sleep, reported moderate strength of evidence that MBIs significantly improved sleep quality compared with nonspecific active controls [75]. A review investigating MBI’s effect on populations with insomnia revealed MBIs significantly improved sleep quality, sleep onset latency, and sleep efficiency [76].

It is important to note that LC is not a psychological illness and authors do not propose that lifestyle interventions, such as MBIs, are intended to treat “the psychological and social origins of the disease” [77], as have been harmfully proposed in earlier models of infection-associated chronic illnesses [78]. The literature does suggest; however, that MBIs can induce neurobiological changes that may be beneficial, including increased cortical thickness, reduced amygdala reactivity, improved brain connectivity and neurotransmitter concentrations that result in improved emotional regulation, cognitive function, and stress resilience [79] [80]. For instance, MBIs may support patients with LC in fostering psychological resilience [81] which plays an important role in skillfully coping with a life-altering chronic disease to gain a sense of well-being even in the face of stress and illness.

This review of literature is important and may assist in integrating several treatments that may be relevant to LC; however, limitations of this case report need to be highlighted. Case reports are unable to link cause with effect, hence, hence we cannot say whether the observed benefits are attributed to the interventions or external factors, such as placebo effect or natural disease progression. Moreover, it is difficult to determine the most impactful intervention component or components when evaluating multimodal treatments. We cannot generalize results to other patients with LC and co-morbidities. Given the lack of scientific knowledge regarding vaccine-related injuries, we are unable to differentiate between LC symptoms and the vaccine related injury and its long-term effects. The strengths of this case report include the high degree of descriptive power, acknowledgment and incorporation of the patient’s perspective, and the development of an etiologic and clinical model of care to be tested in future, larger trials.

## 6. Conclusion

An all-unifying, pathophysiologic underlying mechanism does not yet exist for LC [1] [2] [7]. Given the complexities of this disease, multiple treatment strands, positioned from a whole person health model, that provide multi-component approaches to care are likely ideal treatment [82]. These clinical observations suggest that the synergistic effects of ketogenic metabolic therapy, combined with group health coaching sessions to entrain and stabilize circadian rhythm and teach mindfulness-based practices, may target multiple proposed mechanisms underlying LC, thus, reducing symptom burden and improving quality of life. Findings support future research to optimize and evaluate multimodal nutritional and lifestyle interventions for LC.

## Figures and Tables

**Table 1.** Diagnostic assessments and results.

Test	Date (Months from Last Infection in August 2023)	Findings/Interpretations
Lean Test	10/2023 (2 month)	Heart rate increased from 66 beats per minute (bpm) when sitting to 106 bpm during the first minute of standing, and increased to 124 bpm by 10 minutes.
Transthoracic Echocardiogram	11/2023 (3 months)	All normal findings. The left/ right ventricular chamber and atrium chamber size was normal. No ventricular hypertrophy. No wall motion abnormalities seen. Normal left/right ventricular systolic function. Normal diastolic filling.
Pelvic Ultrasound Examination	11/2023 (3 months)	Normal findings. Unremarkable appearance of the uterine endometrial canal echocomplex. Posterior uterine body serosal margin myometrial masses compatible leiomyoma. Normal ultrasonographic appearance of the left ovary
Autonomic Testing: Tilt Table	1/2024 (4 months)	Beat-to-beat blood pressure and heart rate response to tilt table showed a stable blood pressure with an excessive heart rate response (68 bpm supine to 130 bpm on tilt). Symptoms during head-up-tilt included dizziness, room spinning, chest pressure, head pressure, headache, feet hurting, blood pooling and feet turning purple.
Autonomic Testing: Quantitative Sudomotor Autonomic Reflex Testing	1/2024 (4 months)	Beat-to-beat blood pressure to the Valsalva maneuver showed normal blood pressure variability. QSWEAT responses were normal at the forearm, proximal leg, distal leg and foot.
Serum Tryptase	1/2024 (4 months)	5.5 ug/L (≤10.9 ug/L)
Serum Ferritin	1/2024 (4 months)	13 ng/mL (50 - 240 ng/mL)
Urine prostaglandin D	1/2024 (4 months)	53 pg/mL mg (35 - 115 pg/mL)
Skin Biopsy	2/2024 (5 months)	Normal right upper thigh fiber density at 7.7; normal right lower thigh density at 4.6, and right ankle density significantly decreased at 1.8
Stool	2/2024 (5 months)	Akkermansia muciniphila = ND (1.0 - 8.2e6) Firmicutes = 2.9e11 (5.7e10 - 3.0e11) Enterococcus faecalis = 4.13e4 (< 1.00e4) Streptococcus spp. = 3.93e3 (< 1.00e3) Secretory IgA = 487 (510 - 2010 ug/g) Zonulin = 185 H (<175 ng/g)

*Notes*. Not detectable = ND; Reference range in ().

**Table 2.** Symptom progression following KMT and lifestyle intervention.

Assessment Tool	Administered By	Condition/Variable Assessed	Dec 2023	June 2024 1-Month Post Intervention	Sept. 2024 4-Month Post Intervention	Dec 2024 7-Month Post Intervention	MCID Thresholds
COMPASS-31	Long COVID clinic	Dysautonomia		31		19	MCID not available; >16.4 indicates ANS dysfunction [17]
DASI-METS*	Physical Therapy clinic	Physical activity	3.13	4.62	5.02	5.62	½ Standard Deviation [18]
MIDAS*	Long COVID clinic	Migraine Disability	60	20	12		4.5 points [16]
MMCR *	Long COVID clinic	Dyspnea	Grade 3	Grade 4	Grade 2	Grade 1	1 point [14]
MFIS – Total*	Long COVID clinic	Fatigue	57	59	48	30	13.80 [13] [15]
MFIS Physical			29	30	29	24	6.20
MFIS – Cognitive*			24	22	15	4	6.80
MFIS – Psychosocial			4	7	4	2	MCID not available
GAD-7	Long COVID clinic	Anxiety	2	0	1	0	4 points [19]
PHQ-9	Long COVID clinic	Depression	2	0	2	0	3 points [20]
Blood Pressure	Long COVID clinic		95/56	125/83	110/69	125/70	
Pulse	Long COVID clinic		109	96	74	72	
Body Mass Index	Long COVID clinic		23.04	20.70	20.36	20.18	

*Notes*. COMPASS-31 = Composite Autonomic Symptom Score, >20 suggests moderate-to-severe autonomic dysfunction; DASI = Duke Activity Status Index-METS, higher score indicates higher functional status; MIDAS = Migraine Disability Assessment Questionnaire, 11-20 indicates moderate disability and >21 indicates severe disability; mMCR = Modified Medical Research Council Dyspnea Scale, higher score indicates higher severity; MFIS = Modified Fatigue Impact Scale, higher score indicates greater fatigue. GAD-7 = Generalized Anxiety Disorder, >8 probable case of generalized anxiety disorder; PHQ-9 = Patient Health Questionnaire, >10 probable case of major depressive disorder.

* = indicates improvement in symptoms meets the threshold of minimal clinically important difference.

**Table 3.** Three core components of enable your healing educational program.

Nutritional Therapy	
Macronutrient Distribution of Daily Food Intake	60 - 80 grams of protein
	100 or more grams of fat (from broth and supplemented by grass-fed, grass finished organic ghee, beef tallow, or A2A2 butter, if tolerated)
	15 - 20 grams or fewer of carbohydrate
	*Modifications were made based patients’ need to increase, maintain, or decrease weight*.
	Energy intake was approximately 70% fat, 25% protein, and 5% carbohydrate.
Low Levels of Plant Toxins	Low levels of lectins, oxalates, and endocrine-disrupting glycoalkaloids, such as solanine and phytoestrogens
Eliminate	Sugar and starch
Daily Supplements	Benfotiamine -Fat-soluble form of Vitamin B1 (Starting with 250 mg/day and titrating up, as tolerated, to 2000 mg/day)
	Other Vitamin Bs and Vitamin C (as clinically relevant)
	Two tablespoons of cod liver oil
Daily Supplementation of Electrolytes	Magnesium citrate/malate (3000 mg)
	Potassium citrate (3000 mg)
	Sodium bicarbonate (1200 mg)
	Sodium chloride (1000 mg).
Two to Three Meals per Day	Four to six cups of homemade meat broth daily with cooked vegetables.
Entrain the Circadian Rhythm	

	Exposure to bright morning light
	Reduced blue light in evening
	Intermittent fasting
	Time restricted eating
	Consistent routine of waking, eating, and sleeping every day
Mindfulness-Based Strategies	

	Mindful breathing techniques
	Non-reactive awareness of negative thoughts
	Reframing negative thoughts
	Body Scans
	Mindful Eating
